# Application of statistical machine learning in biomarker selection

**DOI:** 10.1038/s41598-023-45323-9

**Published:** 2023-10-26

**Authors:** Ritwik Vashistha, Zubdahe Noor, Shibasish Dasgupta, Jie Pu, Shibing Deng

**Affiliations:** 1 Department of Statistics and Data Sciences, The University of Texas at Austin, Austin, TX USA; 2 Pfizer Research and Development, Pfizer Healthcare India Private Limited, Chennai, India; 3 Chennai Mathematical Institute, Chennai, India; 4 Pfizer Research and Development, Pfizer, New York, NY USA

**Keywords:** Biomarkers, Prognostic markers

## Abstract

In the recent JAVELIN Bladder 100 phase 3 trial, avelumab plus best supportive care significantly prolonged overall survival relative to best supportive care alone as first-line maintenance therapy following first-line platinum-based chemotherapy in patients with advanced urothelial cancer (aUC). Discovering biomarkers using genomic profiling to understand potential patient heterogeneity is essential to help improve patient care with precision medicine. For the JAVELIN Bladder 100 trial, it is unclear which variable selection methods can most reliably identify biomarkers to inform patient care because the dataset is characterized by high collinearity and low signal. The aim of this paper was to evaluate available selection methods and their ability to discover prognostic and predictive biomarkers in patients with aUC receiving first-line maintenance therapy. A simulation study evaluated the performance of popular variable selection approaches for high-dimensional data including penalized regression models, random survival forests, and Bayesian variable selection methods. For Bayesian variable selection methods, a modified Bayesian Information Criterion (BIC) thresholding rule was proposed in addition to the traditional BIC thresholding rule. These methods were applied to the JAVELIN Bladder 100 dataset to investigate potential biomarkers associated with survival benefit. Results from the simulations demonstrated the strengths and limitations of the different methods. The variable selection methods demonstrated low false discovery rates under different conditions. However, their performance declined in the presence of high collinearity. Using the JAVELIN Bladder 100 data, we identified some potentially significant biomarkers across multiple models. Several lasso-related methods were able to identify potentially biologically meaningful variables in the trial. Some variable selection methods (such as stochastic search variable selection and random survival forest) may not be well suited to this type of data due to the presence of extreme collinearity and low signal. Future research should explore novel variable selection methods that may be more suitable for identifying prognostic and predictive biomarkers in this population.

**Trial registration:** ClinicalTrials.gov Identifier: NCT02603432.

## Introduction

Immune checkpoint inhibitors are established treatments for locally advanced or metastatic urothelial cancer (aUC). Although mechanisms underlying anticancer immunity and immune checkpoint inhibition have been studied extensively, prospective use of biomarkers to identify patients who are most likely to obtain long-term durable benefits from these agents remains unrealized, in part due to variability in assay platform and interpretation across studies. A recent trial (JAVELIN Bladder 100, NCT02603432) showed that the addition of avelumab as first-line maintenance therapy to best supportive care (BSC) significantly prolonged overall survival (OS) compared with BSC alone and established avelumab as a new first-line standard-of-care treatment for aUC^1^. The isolation of avelumab through randomization as the only active treatment covariate in the maintenance setting for aUC provides a unique opportunity to investigate biomarkers that are associated with survival benefit^2^. In particular, the study provides an opportunity to evaluate various methodologies with respect to selecting biomarkers among various candidates, estimating the effects of biomarkers, and combining multiple biomarkers into accurate models.

In published literature, various models have been proposed to address these challenges. For low-dimensional data, the Cox proportional hazards model is the most popular method to study associations of biomarkers with time-to-event endpoints^3^. However, in the context of high-dimensional data (number of biomarkers > number of observations) or in the presence of severe collinearity in the data, the proportional hazards model may not be suitable. Various penalized regression methods have been proposed to overcome these hurdles, among which ridge, lasso (least absolute shrinkage and selection operator), and elastic net are most popular^4^. As an alternative to penalized regression methods, Bayesian methods have also been proposed for variable selection. The main advantage of the Bayesian framework for variable selection is that it allows the incorporation of any prior information regarding the data into the model in addition to transparent quantification of uncertainty. Work by Park and Casella^5^ and Li and Lin^6^ has shown that the frequentist approaches mentioned previously can be outperformed by Bayesian variable selection methods. Various choices of prior distributions have been proposed in the literature for variable selection; however, one type of priors, “spike and slab,” has gained widespread attention due to its intuitive nature and ease of implementation. In the context of survival analysis, Tang et al.^7^ introduced a double-exponential (DE) spike-and-slab prior distribution that was successfully utilized to analyze genes associated with breast cancer in a Dutch dataset. Subsequently, the authors extended their work and proposed group spike-and-slab lasso Cox (gsslasso Cox) to conduct variable selection by incorporating group structures into the model. Tree-based methods have also been proposed as a flexible alternative to the Cox proportional hazards model for modeling survival time and variable selection. In particular, the random survival forest (RSF) has been developed to identify significant covariates and their interactions^8^. Its main advantage over other methods is that it can model complex nonlinear and high-dimensional survival data without strong assumptions regarding the data-generating process.

The main objective of this study was to examine biomarkers associated with survival benefit in the JAVELIN Bladder 100 aUC population using popular variable selection approaches for high-dimensional data and to evaluate variable selection methods using both simulation and the existing biological understanding of aUC biomarkers. In Section “Methods”, we describe various methods that were studied as part of the project and a simulation study conducted to assess the performance of various methods. We also introduce a modified thresholding rule based on Bayesian information criterion (BIC) to select variables based on the posterior estimates of the parameters. In Section “Results”, we present the results obtained by implementing variable selection methods on simulated data and the JAVELIN Bladder 100 dataset. Concluding remarks are provided in Section “Discussion”.

## Methods

### Cox proportional hazards model

In survival analysis, the dataset is usually in the form $({T}_{i}, {\delta }_{i}, {x}_{i})$, where ${T}_{i}$ is the observed time (either failure time or censored time), ${\delta }_{i} \in \{\mathrm{0,1}\}$ is the censoring indicator for an event ${\delta }_{i}=1$ in the case of a failure or death or ${\delta }_{i}=0$ if the observation is censored, and ${x}_{i}$ denotes a *p* dimensional vector of the observed covariates of the *i*th individual. The Cox proportional hazards model^3^ is the most popular method for studying the relationship between observed survival response and explanatory variables. It assumes that $\lambda \left(t|x\right),$ the hazard at time *t* given the vector of explanatory variables $X$, takes the form:

$$\lambda \left(t|X\right)={\lambda }_{0}(t)\mathrm{exp}({\beta }^{T}X)$$

where ${\lambda }_{0}(t)$ is the baseline hazard function, and $X$ and *β* are the vectors of explanatory variables and coefficients, respectively. Here, ${\beta }^{T}x$ is the linear predictor and is also called the risk score. We can estimate the parameter *β* in the model without specification of ${\lambda }_{0}(t)$ by maximizing the partial log-likelihood:

$$pl\left(\beta \right)=\sum_{i=1}^{n}{\delta }_{i}log\left(\frac{\mathrm{exp}({\beta }^{T}{X}_{i})}{{\sum }_{{i}{\prime}\in R({t}_{i})}\mathrm{exp}({\beta }^{T}{X}_{{i}{\prime}})}\right)$$

Here, $R({t}_{i})$ denotes the risk set at time ${t}_{i}$, which contains all subjects who are at risk of an event.

For low-dimensional data, the Cox proportional hazards model can help to understand the relationship between covariates and observed survival response. However, for high-dimensional data, this model fails to be identifiable, or in presence of severe collinearity, regression coefficient estimates $\widehat{\beta }$ fail to converge. Several methods have been proposed to handle such cases, including penalized regression models.

### Penalized regression methods

#### Lasso

Lasso is a regularization method that has L1 Norm as its penalty^9^. Here, coefficients are estimated by minimizing the penalized negative log partial-likelihood:

$$Q\left(\beta \right)=-pl\left(\beta \right)+\lambda \sum_{j=1}^{p}|{\beta }_{j}|$$

where *λ* is the regularization or shrinkage parameter. The estimation of parameters *β* depends on the value of *λ*: a larger value of *λ* implies a higher number of non-zero regression coefficient estimates. We used a 10-fold cross-validation procedure with grid search to find an ‘optimal’ value of *λ*. This procedure was implemented using the glmnet package in R.

#### Elastic net

Zou and Hastie^10^ showed that if a group contains variables with very high pairwise correlations, the lasso tends to randomly select only one variable from the group. To address this issue and other limitations of lasso, they proposed elastic net—a penalized regression method where the penalty is a convex combination of the L1 Norm and the L2 Norm. The presence of an additional L2 Norm term in the penalty makes it possible to promote a grouping effect, thereby removing the limitation of the number of selected variables. Here, coefficients are estimated by minimizing the penalized negative log partial-likelihood:

$$Q\left(\beta \right)= -pl\left(\beta \right)+\lambda (\alpha \sum_{j=1}^{p}\left|{\beta }_{j}\right|+(1-\alpha )\sum_{j=1}^{p}{\left|{\beta }_{j}\right|}^{2})$$

The elastic net penalty is controlled by mixing parameter *α* to bridge the gap between the lasso regression (*α* = 1) and ridge regression (*α* = 0). The parameters (*λ*, *α*) can be estimated using cross-validation with grid search. Because the elastic net has two tuning parameters, we cross-validated on a two-dimensional surface. We first selected a value of *α* from a grid of values, then, for each *α*, we selected a value of *λ* using 10-fold cross-validation.

#### Adaptive lasso

The adaptive lasso introduces a variable-specific weight ${w}_{j}$ into the lasso penalty^11^. The main objective is to penalize larger coefficients less than smaller coefficients to reduce the bias of penalized coefficient estimates found using lasso. The penalized negative log-likelihood is given by:

$$Q\left(\beta \right)=-pl\left(\beta \right)+\lambda \sum_{j=1}^{p}{w}_{j}\left|{\beta }_{j}\right|$$

The variable-specific weights ${w}_{j}$ are of the form $\frac{1}{\left|{\beta }_{j}\right|}$, with $\widetilde{{\beta }_{j}}, j=\mathrm{1,2},\dots p,$ being the solutions of an initial estimation. We considered $\widetilde{{\beta }_{j}}$ to be the ridge estimates found in our data. The optimal value of the parameter *λ* can be found by performing cross-validation as per lasso.

### Random survival forest

RSF is a nonparametric method that has been proposed for modeling survival data^8^. It combines the ideas of bootstrap aggregation and random selection of variables. In our work, survival trees were built according to the parameters recommended by the authors^8^ in the case of high-dimensional data, using the randomForestSRC package in R.

We also performed variable selection using the minimal depth method proposed by Ishwaran et al.^12^. This is a simple and robust method for selecting variables from high-dimensional survival data. Minimal depth evaluates the predictiveness of a variable by its depth in relation to the root node of a tree. The idea can be understood more precisely by defining a maximal subtree for a variable *v,* such that it is the largest subtree whose root node is split using *v* and no other parent node of the subtree is split using *v.* The shortest distance from the root of the tree to the nearest maximal subtree of *v* is the minimal depth of *v*.

### Bayesian variable selection methods

#### Stochastic search variable selection

Stochastic search variable selection (SSVS)^13^ was proposed for variable selection in the context of linear regression. In SSVS, the coefficients *βj* are assumed to follow a suitable Gaussian mixture prior, which induces a positive prior probability on the hypothesis *H*_0_: *β*_*k*_ = 0. Such prior distributions, which are a mixture of two continuous distributions and imply high probability close to zero, are referred as “spike-and-slab” priors. The mathematical formulation of the SSVS prior setup is the following:

$${\beta }_{j}|{\gamma }_{j}=\sim \left(1-{\gamma }_{j}\right)N\left(0,{\tau }_{j}^{2}\right)+{\gamma }_{j}N(0,{c}_{j}^{2}{\tau }_{j}^{2})$$

$${\gamma }_{j}|{p}_{j}\sim Bernoulli({p}_{j})$$

$${p}_{j}\sim Uniform(\mathrm{0,1})$$

Here, ${\gamma }_{j}\in [\mathrm{0,1}]$ acts as a latent variable that facilitates the analysis of performing variable selection. The parameter ${p}_{j}$ can be thought of as the prior probability that ${\beta }_{j}$ is non-zero or that ${X}_{j}$ should be included in the model. The parameters ${\tau }_{j}$ and ${c}_{j}$ can be thought of as tuning parameters that are data dependent. ${\tau }_{j}$ is set to be small so that if ${\gamma }_{j}=0$, ${\beta }_{j}$ can be estimated by 0, while ${c}_{j}$ is set to be large to ensure that a non-zero estimate of ${\beta }_{j}$ can be included in the final model.

We fixed ${c}_{j}$ to be a relatively large value, 1⁄τ_j_, and identified optimal values of ${\tau }_{j}$ using 10-fold cross-validation with grid search.

#### Spike-and-slab lasso Cox

Spike-and-slab lasso (sslasso)^14^ was proposed to integrate two popular methods—lasso and Bayesian spike-and-slab models—into one unifying framework. This method has also been extended for the Cox proportional hazards model to perform variable selection in survival analysis. The sslasso Cox model^7^ was developed by extending the DE prior into the spike-and-slab model because lasso can be expressed as a hierarchical model with DE prior on the coefficients. The mathematical formulation of the prior setup is the following:

$${\beta }_{j}|{\gamma }_{j}\sim \left(1-{\gamma }_{j}\right)DE\left(0,{s}_{0}\right)+{\gamma }_{j}DE(0,{s}_{1})$$

$${\gamma }_{j}|{p}_{j}\sim Bernoulli({p}_{j})$$

$${p}_{j}\sim Uniform(\mathrm{0,1})$$

where the preset scale value *s*_0_ is chosen to be small to induce strong shrinkage on estimation whereas *s*_1_ is chosen to be large to induce weak shrinkage on estimation. The R package BhGLM has been developed to implement the sslasso Cox prior formulation. To find optimal parameters, Tang et al.^7^ suggested to set the slab scale *s*_1_ to be a relatively large value (e.g., 1) and use cross-validation to find an optimal value of *s*_0_.

#### Group spike-and-slab lasso Cox

Group structure can also be incorporated into the sslasso model by assigning a group-specific Bernoulli distribution for the indicator variables^15^. Suppose there are *K* groups with ${m}_{k}$ variables each in the group. For a coefficient, ${\beta }_{{k}_{j}}$ in a group *k*, where *k* = 1*,* 2*….K* and $j=1, 2, \dots , {m}_{k}$, the mathematical formulation is given by:

$${\beta }_{{k}_{j}}|{\gamma }_{{k}_{j}}\sim \left(1-{\gamma }_{{k}_{j}}\right)DE\left(0,{s}_{0}\right)+{\gamma }_{{k}_{j}}DE(0,{s}_{1})$$

$${\gamma }_{{k}_{j}}|{p}_{k}\sim Bernoulli({p}_{k})$$

$${p}_{k}\sim beta(a,b)$$

If group *k* includes important predictors, the parameter ${p}_{k}$ will be estimated to be relatively large, implying that other predictors in the group are likely to be important. For the probability parameters, a beta prior is adopted, which yields the uniform hyperprior ${p}_{k}\sim U(\mathrm{0,1})$, if $a=b=1$.

For all methods, decision rules to determine hyperparameters are described in the Appendix.

### Simulation study

During exploratory data analysis, it was observed that the data were characterized by extreme collinearity and were sparse in nature. To assess the ability of various methods to detect the true variables in the presence of these issues, a simulation study was conducted. The simulated data (n = 450 and *p* = 200, where n is the number of observations, and *p* is the number of variables) was created by varying the number of true variables in the model, their effect size (relative risk reduction of a one-unit increase in the value of variable), and the type of correlation structure in explanatory variables. The survival time was generated from exponential distribution.

#### Simulation settings

Following the simulation study conducted by Tibshirani^9^ and after understanding the structure of our data, we randomly generated blocks of correlated variables from the standard normal distribution with an autoregressive correlation structure, i.e., with homogeneous unit variances and with correlation ($\rho$) declining exponentially within blocks: ${\sigma }_{ij}^{2}={\rho }^{|i-j|}$. We considered $\rho =0.9$ and block size = 50 in the simulation study. The number of true biomarkers in the model (q) was 5 or 10. To generate survival time, we assumed that median survival time was 4 years and considered two values of *β* in the simulations: ${\beta }_{LOW}$ and ${\beta }_{HIGH}$, where:

- ${\beta }_{LOW}$: coefficients of true biomarkers between − 0.4 and − 0.1, and 0 otherwise.
- ${\beta }_{HIGH}$: coefficients of true biomarkers between − 1 and − 0.5, and 0 otherwise.

The censoring time was generated from the exponential distribution where the parameter c was chosen to keep the censoring rate near 50%. In total, 4 designs with an autoregressive correlation structure were created as part of the simulation study. Information about simulated datasets is summarized in Table 1.

**Table 1:** Simulated data.

Design	No. of true variables	Magnitude of coefficients	Correlation structure
Design 1	5	Low	Autoregressive
Design 2	5	High	Autoregressive
Design 3	10	Low	Autoregressive
Design 4	10	High	Autoregressive

#### Measures for evaluation of results

The data were generated randomly under each design 100 times. The variable selection capability of different models was judged by computing three operating characteristics based on the parameter estimates: true positive rate (TPR or sensitivity), true negative rate (TNR or specificity), and false positive rate (FPR). The formulas for these operating measures are as follows:

$$TPR=\frac{Number \, of  \, true  \, variables  \, correctly  \, entered  \, into  \, the  \, model}{Total  \, number  \, of  \, variables}$$

$$TNR=\frac{Number  \, of \,  irrelevant  \, variables  \, correctly  \, excluded \,  from  \, the  \, model}{Total  \, number  \, of  \, variables}$$

$$FPR=\frac{Number \,  of  \,  irrelevant \,  variables  \, mistakenly  \, entered \,  into \,  the  \, model}{Total  \, number  \, of  \, variables}$$

For penalized methods, variable selection was performed using non-zero coefficient estimates. In RSF, variables were selected using variable importance through minimal depth procedure. However, for Bayesian methods, variables were selected using three alternative rules because posterior estimates are non-zero:

- Confidence interval (CI) rule: variables whose $\left(1-\alpha \right)\%$ CI for coefficient estimates $[\widehat{\beta }-{Z}_{\alpha /2}*SE\left(\widehat{\beta }\right), \widehat{\beta }+{Z}_{\alpha /2}*SE\left(\widehat{\beta }\right) ]$ does not contain 0 are selected by the model. Here, ${Z}_{\alpha /2}$ is the critical value when the right-tailed area under a standard normal distribution is given by α/2, i.e.,
  $$P\left(Z>{Z}_{\alpha }\right)=\alpha $$
  where $Z\sim N(\mathrm{0,1})$. We considered $\alpha =0.25, 0.1,\;or\; 0.05$ for calculating the CIs.
- BIC thresholding rule: Lee, Chakraborty, and Sun ^16^ proposed the BIC thresholding rule for variable selection. Here, the absolute posterior estimates of ${\beta }_{j}$ are initially arranged in descending order, and BIC values are computed in a stepwise manner by sequentially adding important covariates. The formula for BIC with $j$ largest ${\beta }_{j}$ is written as:
  $${BIC}_{j}= -2\left(l\left({\widehat{\beta }}_{\left(1:j\right)}\right)-l\left(0\right)\right)+jlog(n)$$
  where n is the number of observations, $l\left({\widehat{\beta }}_{\left(1:j\right)}\right)$ denotes the maximized log-likelihood under a model that includes the variables corresponding to the first $j$ largest absolute posterior estimates ${\widehat{\beta }}_{j}$ s given by ${\widehat{\beta }}_{\left(1:j\right)}$. $l\left(0\right)$ denotes the log-likelihood under the null model. ${BIC}_{j}$ s are computed by sequentially adding important variables and the best model is chosen where its minimum occurs. To shortlist variables before computation of BIC, we considered the top 50 variables with the largest non-zero absolute posterior estimates (j = 50).
- Modified BIC thresholding rule: We also considered a modified version of the BIC thresholding rule above. We proceeded as follows:
  1. Select the top 50 variables with the largest non-zero absolute posterior estimates.
  2. Consider the variable with the highest coefficient and include it in the model.
  3. Include a variable in the model from the list of remaining variables for which BIC is minimum.
  4. Continue adding variables and computing BIC as in Step 3 until there are no remaining variables to be considered.
  5. Consider the model with minimum BIC value as the final model.

Our modified approach is likely to be less conservative in selecting variables than the original BIC approach. However, the computation time increases due to additional comparisons.

### Ethics approval and consent to participate

The trial was conducted in accordance with the ethics principles of the Declaration of Helsinki and with the Good Clinical Practice guidelines defined by the International Council for Harmonization. All the patients provided written informed consent. The experimental protocol and amendments were approved by Pfizer.

## Results

### Simulated data

Results were obtained for all 100 replications of the four designs that were considered in the study, and average measures of TPR, TNR, and FPR are shown in Tables 2, 3, 4 and 5 for different methods. In our simulation study, we considered penalized, RSF, and Bayesian spike-and-slab models for comparison. Optimal parameters were found using the cross-validation procedure mentioned in the methods section for each model. Table 2 shows that elastic net had the highest TPR (sensitivity) among penalized regression methods for different designs, followed by lasso, adaptive lasso, and RSF. However, RSF, followed by lasso and elastic net, had high FPRs and consequently low specificity compared with adaptive lasso. With RSF, a huge number of variables were chosen, resulting in an extremely high FPR and poor performance across different designs. However, adaptive lasso tried to achieve a balance between TPRs and FPRs and had a significantly lower FPR than RSF and the other two penalized regression methods. The decrease in the value of FPR in adaptive lasso came at the cost of the number of true discoveries and results in a relatively low sensitivity compared with the other models.

**Table 2:** Simulation results for penalized regression methods and RSF.

Design	Lasso			Elastic net			Adaptive lasso			RSF		
	TPR	TNR	FPR	TPR	TNR	FPR	TPR	TNR	FPR	TPR	TNR	FPR
Design 1	0.87	0.91	0.09	0.93	0.89	0.11	0.87	0.94	0.06	0.79	0.24	0.76
Design 2	0.96	0.90	0.10	1.00	0.90	0.10	0.85	0.97	0.03	0.82	0.19	0.80
Design 3	0.74	0.88	0.12	0.83	0.83	0.17	0.66	0.93	0.07	0.63	0.23	0.77
Design 4	0.99	0.85	0.15	1.00	0.84	0.16	0.91	0.93	0.07	0.79	0.21	0.76

*FPR* false positive rate, *lasso* least absolute shrinkage and selection operator, *RSF* random survival forest, *TNR* true negative rate, *TPR* true positive rate.

**Table 3:** Simulation results for Bayesian methods with BIC rule for variable selection.

Design	gsslasso-BIC			gsslasso-modified BIC			SSVS-BIC			SSVS-modified BIC		
	TPR	TNR	FPR	TPR	TNR	FPR	TPR	TNR	FPR	TPR	TNR	FPR
Design 1	0.63	0.99	0.01	0.70	0.99	0.01	0.12	0.99	0.01	0.50	0.98	0.02
Design 2	0.81	1.00	0.89	0.89	0.99	0.01	0.59	0.97	0.03	0.89	0.99	0.01
Design 3	0.42	0.98	0.44	0.44	0.98	0.02	0.15	0.97	0.03	0.31	0.97	0.03
Design 4	0.93	0.99	0.95	0.95	0.99	0.01	0.85	0.95	0.05	0.88	0.98	0.02

*BIC* Bayesian information criterion, *FPR* false positive rate, *gsslasso* group spike-and-slab least absolute shrinkage and selection operator, *SVSS* stochastic search variable selection, *TNR* true negative rate, *TPR* true positive rate.

**Table 4:** Simulation results for gsslasso Cox prior with CI rule for variable selection.

Design	75% CI rule			90% CI rule			95% CI rule		
	TPR	TNR	FPR	TPR	TNR	FPR	TPR	TNR	FPR
Design 1	0.75	0.97	0.03	0.76	0.97	0.03	0.64	0.99	0.01
Design 2	0.92	0.98	0.02	0.93	0.97	0.03	0.89	0.99	0.01
Design 3	0.55	0.96	0.04	0.57	0.95	0.05	0.41	0.98	0.02
Design 4	0.97	0.97	0.03	0.98	0.96	0.04	0.94	0.99	0.01

*CI* confidence interval, *FPR* false positive rate, *gsslasso* group spike-and-slab least absolute shrinkage and selection operator, *TNR* true negative rate, *TPR* true positive rate.

**Table 5:** Simulation results for SSVS prior with CI rule for variable selection.

Design	75% CI rule			90% CI rule			95% CI rule		
	TPR	TNR	FPR	TPR	TNR	FPR	TPR	TNR	FPR
Design 1	0.76	0.65	0.35	0.74	0.63	0.37	0.66	0.80	0.20
Design 2	0.92	0.75	0.25	0.94	0.73	0.27	0.91	0.84	0.16
Design 3	0.56	0.69	0.31	0.58	0.68	0.32	0.47	0.81	0.19
Design 4	0.91	0.92	0.08	0.92	0.90	0.10	0.87	0.93	0.07

*CI* confidence interval, *FPR* false positive rate, *SVSS* stochastic search variable selection, *TNR* true negative rate, *TPR* true positive rate.

The results for Bayesian methods are summarized in Tables 3, 4, and 5. In Table 3, we compare a modified BIC approach for selection with the original BIC approach for both Bayesian models. Our modified approach performed better than the original approach for both Bayesian models. Among the two Bayesian models, gsslasso performed the best and showed higher sensitivity and specificity than the SSVS model.

In the case of the CI rule for variable selection, we see from Table 4 that the 90% CI rule performed better than the other choices for gsslasso Cox prior owing to its higher sensitivity. However, for the SSVS model, as shown in Table 5, the 95% CI rule was the most appropriate choice owing to the high values of FPRs in the other two choices for CI. Overall, however, SSVS performed less well than other Bayesian methods.

From our simulation study, the adaptive lasso and gsslasso Cox were concluded to be the most appropriate models for variable selection from the two classes of models. Both had moderate sensitivity and high specificity. Additionally, the gsslasso Cox model had the advantage of incorporating the group structure into the model. Because keeping the false discovery rate low is highly important in a clinical setting, both methods were concluded to be more appropriate for variable selection in the presence of high collinearity than their counterparts.

### Real data

We assessed the performance of the various methods discussed in Section “Results” on using data from a recent phase 3 trial: JAVELIN Bladder 100 (NCT02603432). The data contained information from 688 patients for 189 variables. Among the 189 variables, two were related to OS outcome (OS_EVENT and OS), one was treatment arm (TRT01P1, “avelumab + BSC” is the treatment arm and “BSC” is the control arm), four were baseline patient characteristics (SEX, AGE, STRATI11 [best response to first-line chemotherapy], and STRATI21 [metastatic disease site at first-line chemotherapy]), and the remaining 182 were biological features (biomarkers) of interest.

There were 344 observations in the treatment group and 344 observations in the control group (Table 6). Of the total of 688 observed times, 320 were uncensored observations (failure times) and 368 (53.4%) were censored observations. However, among the 688 observations, only 429 observations had complete data for all variables (37*.*6% missing observations). Eliminating the missing observations, only 222 observations remained in the treatment group and 207 observations remained in the control group. Of these 429 observed times, 198 were uncensored observations and 231 were censored observations (53.8%). For our analysis, we excluded all missing observations from the data and analyzed only complete data.

**Table 6:** Data description.

	Whole data	Complete data	Missing data (%)
Total no. of observations	688	429	37.64
Treatment group	344	222	35.47
Control group	344	207	39.83
Censored observations	368	231	37.23
Uncensored observations	320	198	38.13

We found that the data (excluding variables related to OS outcome) originally consisted of 5 binary variables and 182 numerical variables. Three new binary variables were created and included in the analysis. Post feature engineering, the data had 8 binary variables and 178 numerical variables. The data were also found to have a high amount of sparsity in some features, with 12 of 186 numeric variables (excluding OS_EVENT and OS from the total 188 variables) having at least 60% “zeroes” in their observations. Although, some of the sparse features had a low number of unique values, they were still considered as numerical variables in our analysis.

In exploratory data analysis, we found that the data had severe multicollinearity. Additionally, the variables had a natural group structure such that 5 groups of variables had varying lengths and some other variables were not part of any group (mentioned in Supplement S2). The variables in one group were found to have “inner correlation” (correlation between themselves) and “outer correlation” (correlation with variables outside their own group).

In summary, the main characteristics of our data are:

1. Severe collinearity among the explanatory variables,
2. High-dimensional data with not enough sample size,
3. Sparsity in the data (8.06%), and
4. High percentage of censored observations (53.8%).

On comparing full data with treatment-only data, we found that the collinearity was more severe for treatment-only data. The percentage of censored observations also increased for treatment-only data (59.4% from 53.8%). However, the sparsity in the data remained similar (7.82% from 8.06%).

We focused both on the full data, which contained information on patients assigned to treatment and control observations, in addition to the data subset containing only the observations of patients who were assigned to treatment. Optimal parameters for all the methods except RSF were found using the cross-validation procedures.

For RSF, we considered the value of parameters recommended by Ishwaran et al.^8^. We observed that RSF gave very poor prediction results and had a high prediction error rate for both full and treatment-only data (46.96% and 46.48%, respectively). Tuning parameters did not reduce the prediction error. We further performed variable selection by shortlisting the top 15 variables with highest variable importance. The results are presented in two tables in the supplement (Tables S1 & S2) that summarize the variables selected by different methods. For Bayesian models, we used only the 90% or 95% CI rule and our modified BIC approach for variable selection after observing their performance in the simulation study.

#### Full data

Table 7 reports the results for the analysis of full data, showing only those variables selected by at least two of the methods considered in our assessment; the full list of selected variables is reported in Table S1. None of the variables were selected by all the models. However, all the penalized models, gsslasso Cox, and RSF selected the treatment variable and thus validated its relevance. Very few variables were selected by both penalized and Bayesian methods. Except for the treatment variable, only “cytopro.effector_memory_CD8.positive_alpha.beta_T_cell,” “IC_PD_L1_Status1,”, and “LM22.Mast_cells_activated” were selected by both classes of methods. All the penalized models selected the variables “cytopro.neutrophil,” “cytopro.effector_memory_RA_-CD8.positive_alpha.beta_T_cell_.TEMRA,” and “STRATI21,” whereas these were not identified as being relevant by Bayesian methods. In contrast, Bayesian methods selected “LM22.T_cells_CD8” and “LM22.NK_- cells_resting,” whereas these were not identified as being relevant by penalized models. These different results highlight a stark contrast between the two classes of methods in selecting variables from the data, making the relevance of these variables unclear. However, the significantly lower false discovery rate of Bayesian methods compared with penalized models favors the Bayesian methods. Furthermore, gsslasso Cox performed better than sslasso Cox due to incorporation of the group structure in the model.

**Table 7:** Results (full data).

Variable	Lasso	Elastic net	Adaptive lasso	SSVS-95% CI	sslasso-90% CI	gsslasso-90% CI	gsslasso-modified BIC	RSF
TRT01P1	Yes	Yes	Yes	No	No	Yes	Yes	No
STRATI21	Yes	Yes	Yes	No	No	No	No	No
Crypto.effector_memory_CD8.positive_alpha.beta_T_cell	Yes	Yes	Yes	No	No	No	No	Yes
Crypto.effector_memory_RA_CD8.positive_alpha.beta_T_cell_.TEMRA	Yes	Yes	Yes	No	No	No	No	Yes
Cytopro.neutrophil	Yes	Yes	Yes	No	No	No	No	Yes
IC_PD_L1_Status1	Yes	Yes	Yes	No	No	Yes	No	No
TMB_pre_chemo	No	Yes	No	No	No	No	No	Yes
LM22.T_cells_regulatory_Tregs	No	Yes	No	No	No	No	No	No
LM22.Mast_cells_activated	No	Yes	No	Yes	Yes	Yes	Yes	Yes
B999001_c11_Epithelium_development	No	Yes	No	No	No	No	No	Yes
Cytopro.CD8.positive_alpha.beta_T_cell	No	Yes	Yes	No	No	No	No	Yes
LM22.NK_cells_resting	No	No	No	Yes	Yes	Yes	Yes	No
LM22.T_cells_CD8	No	No	No	Yes	Yes	No	No	No
LM22.T_cells_gamma_delta	No	No	No	No	No	Yes	Yes	No
Cytopro.CD14.positive_CD16.positive_monocyte	No	No	No	No	No	Yes	No	No

*CI* confidence interval, *gsslasso* group spike-and-slab least absolute shrinkage and selection operator, *lasso* least absolute shrinkage and selection operator, *RSF* random survival forest, *sslasso* spike-and-slab least absolute shrinkage and selection operator, *SVSS* stochastic search variable selection.

#### Treatment-only data

Table 8 reports the results for the analysis of treatment-only data, showing only those variables selected by at least two methods considered in our assessment; the full list of selected variables is reported in Table S2. Results were similar to those observed in the analysis of full data. As observed for the treatment variable in the analysis of full data, “STRATI21” was selected by all of the penalized models and by gsslasso Cox, but not by RSF. All of the penalized models selected the variables “Number_high_affinity_FCGR_alleles” and “cytopro.CD8.positive_alpha.beta_-T_cell,” whereas these were not identified as being relevant by Bayesian methods. In contrast, Bayesian methods selected “HALLMARK_IL2_STAT5_SIGNALING” and “LM22.Mast_cells_activated,” whereas these were not identified as being relevant by penalized models. “TUMOR_CELL_STAINING_analytePDL1” and “cytopro.effector_memory_CD8.positive_alpha.beta_T_cell” were selected by both the penalized and Bayesian models.

**Table 8:** Results (treatment-only data).

Variable	Lasso	Elastic net	Adaptive lasso	SSVS-95% CI	sslasso-90% CI	gsslasso-90% CI	gsslasso-modified BIC	RSF
STRATI21	Yes	Yes	Yes	No	No	Yes	Yes	No
TMB_pre_chemo	Yes	Yes	No	No	No	No	No	Yes
Number_high_affinity_FCGR_alleles1	Yes	Yes	Yes	No	No	No	No	No
cytopro.CD8.positive_alpha.beta_T_cell	Yes	Yes	Yes	No	No	No	No	Yes
cytopro.effector_memory_CD8.positive_alpha.beta_T_cell	Yes	Yes	Yes	No	No	No	No	Yes
TUMOR_CELL_STAINING_analytePDL11	No	Yes	No	No	No	Yes	No	No
B9991001_c11_Epithelium_development	No	Yes	No	No	No	No	No	Yes
B9991003_c15_Skin_development	No	Yes	No	No	No	No	No	Yes
cytopro.effector_memory_RA_CD8.positive_alpha.beta_T_cell_.TEMRA	No	Yes	Yes	No	No	No	No	Yes
cytopro.granulocyte	No	Yes	Yes	No	No	No	No	Yes
cytopro.mast_cell	No	No	Yes	No	No	No	No	Yes
LM22.Mast_cells_activated	No	No	No	Yes	Yes	Yes	Yes	No
HALLMARK_IL2_STAT5_SIGNALING	No	No	No	Yes	Yes	Yes	Yes	No

*CI* confidence interval, *gsslasso* group spike-and-slab least absolute shrinkage and selection operator, *lasso* least absolute shrinkage and selection operator, *RSF* random survival forest, *sslasso* spike-and-slab least absolute shrinkage and selection operator, *SVSS* stochastic search variable selection.

## Discussion

We assessed various methods of variable selection in survival analysis. Our objectives were to find important variables in a recent clinical trial dataset using different methods and to find a suitable method for variable selection. To understand the properties of different methods, we performed a simulation study that reflected key issues present within our data. In particular, we assessed the performance of penalized regression models, RSF, and Bayesian spike-and-slab models in the presence of groups of highly correlated variables with low signals within the data. In the simulation study, we found that RSF selected the highest number of variables, followed by penalized regression methods and Bayesian methods, and had higher sensitivity. However, because of its less restrictive nature in selecting variables, RSF also had a higher FPR than Bayesian methods. Elastic net had the highest sensitivity and the lowest specificity among the classic penalized regression models in all the scenarios. Adaptive lasso aimed to achieve a balance between TPRs and FPRs, and had a significantly lower FPR than lasso and elastic net for a marginal decrease in sensitivity. Among Bayesian methods, the gsslasso Cox model had the best overall performance in all scenarios owing to its extremely high specificity and moderate sensitivity. We also considered various rules for variable selection because posterior estimates of coefficients are not exactly zero, unlike in penalized regression models. In the case of the gsslasso Cox model, we found that the 90% CI rule and our modified BIC approach provided similarly good results. However, for the SSVS model, the CI rule performed poorly in terms of specificity for different designs and was inferior to the BIC approach. Following the simulation study, we applied all the methods studied to clinical trial data but observed poor performance. We assessed the penalized regression models and Bayesian spike-and-slab models in addition to RSF. Results for penalized regression models and Bayesian spike-and-slab models were slightly inconsistent with few variables identified by both classes of models in both full and treatment-only data. The difference in the results made the relevance of the selected variables unclear. However, variables that were selected by both classes of models can be considered potentially significant. Using full data, “TRT01P1,” “IC_PD_L1_Status1,” and “LM22.Mast_cells_activated” were selected by both model classes, and for treatment-only data, “STRATI21” and “TUMOR_CELL_STAINING_analytePDL1” were selected by both model classes. Because Bayesian spike-and-slab models had very low false discovery rates, the probability that common variables were false discoveries is low.

RSF was also applied to the same datasets, but its performance was poor owing to high prediction error. Tuning parameters did not improve this model, but we still performed variable selection using minimal depth procedure. Despite its poor prediction performance, some variables selected using RSF were the same as those selected by other methods.

There remains much room for further research. We only considered main effects in our regression models. A future direction of work may focus on incorporating interaction effects in the regression model in the presence of a high amount of noise in the data.

In conclusion, we assessed various well-known variable selection methods and identified potentially significant variables or biomarkers. However, these methods may not be well suited to analyzing this type of dataset because of the presence of extreme collinearity and low signal. The sslasso model can overcome collinearity owing to its ability to incorporate group structure in the model; however, it does not perform well if low signals are present.

### Supplementary Information

Supplementary Information.

## Data Availability

The data that support the findings of this study are available from Pfizer, but restrictions apply to the availability of these data, which were used under license for the current study, and so are not publicly available. Data are however available from the authors upon reasonable request and with permission of Pfizer. Subject to certain criteria, conditions and exceptions, Pfizer may also provide access to the related individual de-identified participant data. See https://www.pfizer.com/science/clinical-trials/trial-data-and-results for more information.

## Appendix

### Decision rules

We used the following decision rules for determining hyperparameters.

1. Lasso:
  We used a modified version of a 10-fold cross-validation procedure with grid search to find an “optimal value” of *λ*. Generally, in k-fold cross-validation, the data are split randomly into k in approximately equally sized groups. The model is fitted using k-1 of these sets and the omitted set is used to test the model. This procedure is repeated k times, until all groups have been omitted once. The sum or average of the errors (log-partial likelihood) evaluated on each omitted set for all the groups is used to measure model performance. However, where data include heavy censoring or in the case of leave-one-out cross-validation, the partial likelihood equation of the Cox proportional hazards model may become ill-defined and the cross-validation would fail to help in finding an optimal value of the parameter. To tackle this issue, we used the technique proposed by Van Houwelingen et al.^17^ to obtain the log-partial likelihood. The data are split into k parts. The goodness-of-fit estimate for given part *i* and *λ* is:
  $$\widehat{{CV}_{i}}=l\left({\beta }_{-i}\left(\lambda \right)\right) -{l}_{-i}\left({\beta }_{-i}\left(\lambda \right)\right)$$
  where ${l}_{-i}$ is the log-partial likelihood excluding part *i* of the data, and ${\beta }_{-i}$ is the optimal *β* for the non-left-out data, found from maximizing ${l}_{-i}+\lambda {\Vert \beta \Vert }_{1}$. The final total goodness-of-fit estimate, $\widehat{CV}$, is the sum of all $\widehat{{CV}_{i}}$. We chose *λ,* which maximizes $\widehat{CV}$. However, due to randomness in the cross-validation procedure, it is common to obtain different ‘optimal’ values of $\lambda$ and, consequently, different models when the k-fold procedure is performed repeatedly. In this situation, it is difficult to select a final value of *λ*.
  To tackle this issue, we performed 100 iterations of the 10-fold cross-validation procedure with grid search. We selected the most commonly occurring *λ* value and chose the corresponding model to be our final model. This procedure was implemented using the glmnet package in R.
2. Elastic net:
  The parameters (*λ*, *α*) are estimated using cross-validation with grid search. Because the elastic net has two tuning parameters, we cross-validated on a two-dimensional surface. First, we selected a value of *α* from a grid of values, then for each *α*, we selected a value of *λ* using 10-fold cross-validation. As described for lasso, we repeated this procedure 100 times to obtain 100 pairs of (*λ*, *α*) values, and we selected the most commonly occurring (*λ*, *α*) pair as the final value of the parameters.
3. Adaptive lasso:
  The optimal value of the parameter *λ* can be found by performing cross-validation as per lasso.
4. Random survival forest:
  No hyperparameter tuning was performed.
5. Stochastic search variable selection:
  We fixed ${c}_{j}$ to be a relatively larger value, 1⁄τ_j,_ and found the optimal values of parameter ${\tau }_{j}$ using 10-fold cross-validation with a grid search.
6. Spike-and-slab lasso Cox and group spike-and-slab lasso Cox:
  We set the slab scale *s*_1_ to be a relatively large value (e.g., 1) and used cross-validation to find an optimal value of *s*_0_.

## Abbreviations

aUC: Locally advanced or metastatic urothelial cancer
BIC: Bayesian information criterion
BSC: Best supportive care
DE: Double-exponential
FPR: False positive rate
gsslasso Cox: Group spike-and-slab lasso Cox
lasso: Least absolute shrinkage and selection operator
OS: Overall survival
RSF: Random survival forest
sslasso: Spike-and-slab lasso
SSVS: Stochastic search variable selection
TNR: True negative rate
TPR: True positive rate
VIF: Variance inflation factor
