# Author Correction: Application of statistical machine learning in biomarker selection

**DOI:** 10.1038/s41598-024-52345-4

**Published:** 2024-01-25

**Authors:** Ritwik Vashistha, Zubdahe Noor, Shibasish Dasgupta, Jie Pu, Shibing Deng

**Affiliations:** 1 Department of Statistics and Data Sciences, The University of Texas at Austin, Austin, TX USA; 2 Pfizer Research and Development, Pfizer Healthcare India Private Limited, Chennai, India; 3 Chennai Mathematical Institute, Chennai, India; 4 Pfizer Research and Development, Pfizer, New York, NY USA

Correction to: *Scientific Reports* 10.1038/s41598-023-45323-9, published online 26 October 2023

The original version of this Article contained an error in Affiliation 2 and 4

Affiliation 2 was incorrectly given as ‘Global Product Development, Pfizer Healthcare India Private Limited, Chennai, India.’ The correct affiliation is listed below:

Pfizer Research and Development, Pfizer Healthcare India Private Limited, Chennai, India.

and Affiliation 4 was incorrectly given as ‘Global Product Development, Pfizer, New York, NY, USA.’ The correct affiliation is listed below:

Pfizer Research and Development, Pfizer, New York, NY, USA.

Also the Acknowledgements section in the original version of this Article was incomplete.

“The authors thank the patients and their families, investigators, co-investigators, and the study teams at each of the participating centers. We thank Craig Davis, Christian Russel Reyes, and the JAVELIN Bladder 100 study team for their constructive comments that greatly improved the manuscript.”

the acknowledgement now reads:

“The authors thank the patients and their families, investigators, co-investigators, and the study teams at each of the participating centers. We thank Craig Davis, Christian Russel Reyes, and the JAVELIN Bladder 100 study team for their constructive comments that greatly improved the manuscript. We would also like to express our deep gratitude to Dr Minerva Mukhopadhyay, Indian Institute Technology (IIT), Kanpur, India for her invaluable support and insights throughout the research work. Lastly, we thank the peer reviewers and editors for their insightful feedback, which helped to improve the manuscript.”

The original Article has been corrected.

