# Supplementary material for: Application of statistical machine learning in biomarker selection
Source: Sci Rep. 2023 Oct 26;13:18331. doi: 10.1038/s41598-023-45323-9 (PMC10603146; doi:10.1038/s41598-023-45323-9)
Supplement: Supplementary file 1 — Supplementary Information. [file 41598_2023_45323_MOESM1_ESM.docx]

**Supplement**

**Figure 1. VIF plot**


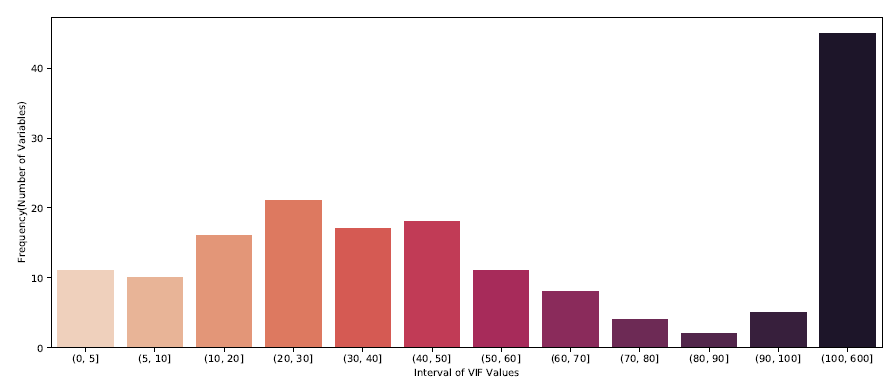


Figure 1: Frequency distribution of VIF values (before the elimination of numerical features whose VIF > 10). VIF, variance inflation factor.

**S2. Groups in data**

Following groups are formed for incorporating the group structure:

• "mutSig.SBS1" - "mutSig.SBS2_13"

• “HALLMARK_HEME_METABOLISM” - “HALLMARK_MYC_TARGETS_V1”

• “LM22.Eosinophils” - “LM22.Neutrophils”

• “B9991001_c3_Immune_response_lymphocytic_and_c7_IFNG_response” - “B9991003_- c17_Glucocorticoid_metabolic_process”

• “cytopro.CD14.low_CD16.positive_monocyte” - “cytopro.stromal cell”

Fifteen variables are not part of any group. Each of those 15 variables are considered as an individual group, so in all there are 21 groups.

Table S1: Results - Full Data

| Variables | Lasso | Elastic net | Adaptive lasso | SSVS-95% CI | sslasso-90% CI | gsslasso-90% CI | gsslasso-90% BIC | RSF |
| --- | --- | --- | --- | --- | --- | --- | --- | --- |
| TRT01P1  STRATI21  Crypto.effector_memory_CD8.positive_alpha.beta_T_cell  Crypto.effector_memory_RA_CD8.positive_alpha.beta_T_cell_.TEMRA.  Cytopro.neutrophil  IC_PD_L1_Status1  FCGR2A.RS1801274.  TMB_pre_chemo  MBIOQLR_analytePDL11  POSITIVE_CELLS_INVASIVE_MARGIN_analyteCD8  LM22.T_cells_regulatory_Tregs  M22.T_cells_follicular_helper  LM22.Mast_cells_activated  B999001_c11_Epithelium_development  cytopro.CD8.positive_alpha.beta_T_cell  mutSig.SBS1  cytopro.mast_cell  cytopro.regulatory_T_cell  POSITIVE_CELLS_TUMOR_CENTER_analyteCD8  LM22.B_cells_naive  B9991003_c15_Skin_development  mutSig.SBS5  LM22.Mast_cells_resting  LM22.Neutrophils  HALLMARK_APICAL_SURFACE  LM22.NK_cells_resting  LM22.T_cells_CD8  LM22.Macrophages_M2  LM22.T_cells_gamma_delta  cytopro.CD14.positive_CD16.positive_monocyte  cytopro.macrophage | Yes  Yes  Yes  Yes  Yes  Yes  No  No  No  No  No  No  No  No  No  No  No  No  No  No  No  No  No  No  No  No  No  No  No  No  No | Yes  Yes  Yes  Yes  Yes  Yes  Yes  Yes  Yes  Yes  Yes  Yes  Yes  Yes  Yes  Yes  No  No  No  No  No  No  No  No  No  No  No  No  No  No  No | Yes  Yes  Yes  Yes  Yes  Yes  No  No  No  No  No  No  No  No  Yes  No  Yes  Yes  No  No  No  No  No  No  No  No  No  No  No  No  No | No  No  No  No  No  No  No  No  No  No  No  No  Yes  No  No  No  No  No  No  No  No  No  No  No  No  Yes  Yes  No  No  No  No | No  No  No  No  No  No  No  No  No  No  No  No  Yes  No  No  No  No  No  No  No  No  No  No  No  No  Yes  Yes  No  No  No  No | Yes  No  No  No  No  Yes  No  No  No  No  No  Yes  No  Yes  Yes  No  No  No  No  No  No  No  No  No  No  Yes  No  Yes  Yes  Yes  Yes | Yes  No  No  No  No  No  No  No  No  No  No  Yes  No  Yes  No  No  No  No  No  No  No  No  No  No  No  Yes  No  No  Yes  No  No | No  No  Yes  Yes  Yes  No  No  Yes  No  No  No  No  Yes  Yes  Yes  Yes  Yes  Yes  Yes  Yes  Yes  Yes  Yes  Yes  Yes  No  No  No  No  No  No |

BIC, Bayesian information criterion; CI, confidence interval; gsslasso, group spike-and-slab least absolute shrinkage and selection operator; lasso, least absolute shrinkage and selection operator; RSF, random survival forest; sslasso, spike-and-slab least absolute shrinkage and selection operator; SVSS, stochastic search variable selection.

Table S2: Results – Treatment Only Data

| Variables | Lasso | Elastic Net | Adaptive Lasso | SSVS-95%CI | SS Lasso-90%CI | GSS Lasso-90%CI | GSS Lasso-BIC | RSF |
| --- | --- | --- | --- | --- | --- | --- | --- | --- |
| STRATI21  TMB_pre_chemo  Number_high_affinity_FCGR_alleles1  cytopro.CD8.positive_alpha.beta_T_cell  cytopro.effector_memory_CD8.positive_alpha.beta_T_cell  FCGR2A.RS1801274.  MBIOQLR_analytePDL11  TUMOR_CELL_STAINING_analytePDL11  POSITIVE_CELLS_TOTAL_AREA_analyteCD8  POSITIVE_CELLS_TUMOR_CENTER_analyteCD8  B9991001_c11_Epithelium_development  B9991003_c15_Skin_development  B9991003_c10_Myogenesis  cytopro.effector_memory_RA_CD8.positive_alpha.beta_T_cell_.TEMRA.  cytopro.granulocyte  cytopro.mature_natural_killer_cell  cytopro.natural_killer_cell  cytopro.mast_cell  cytopro.naive_thymus.derivved_CD4.positive_alpha.beta_T_cell  LM22.Mast_cells_activated  HALLMARK_IL2_STAT5_SIGNALING  HALLMARK_ANDROGEN_RESPONSE  B9991003_c20_Interferon_gamma_response  HALLMARK_APICAL_SURFACE | Yes  Yes  Yes  Yes  Yes  No  No  No  No  No  No  No  No  No  No  No  No  No  No  No  No  No  No  No | Yes  Yes  Yes  Yes  Yes  Yes  Yes  Yes  Yes  Yes  Yes  Yes  Yes  Yes  Yes  Yes  Yes  No  No  No  No  No  No  No | Yes  No  Yes  Yes  Yes  No  No  No  No  No  No  No  No  Yes  Yes  No  No  Yes  Yes  No  No  No  No  No | No  No  No  No  No  No  No  No  No  No  No  No  No  No  No  No  No  No  No  Yes  Yes  No  No  No | No  No  No  No  No  No  No  No  No  No  No  No  No  No  No  No  No  No  No  Yes  Yes  No  No  No | Yes  No  No  No  No  No  No  Yes  No  No  No  No  No  No  No  No  No  No  No  Yes  Yes  Yes  No  No | Yes  No  No  No  No  No  No  No  No  No  No  No  No  No  No  No  No  No  No  Yes  Yes  No  No  No | No  Yes  No  Yes  Yes  No  No  No  No  Yes  Yes  Yes  No  Yes  Yes  Yes  No  Yes  Yes  No  No  No  Yes  Yes |
